# Supplementary material for: Molecular fingerprints resolve affinities of Rhynie chert organic fossils
Source: Nat Commun. 2023 Mar 13;14:1387. doi: 10.1038/s41467-023-37047-1 (PMC10011563; doi:10.1038/s41467-023-37047-1)
Supplement: Supplementary file 1 — Supplementary Information [file 41467_2023_37047_MOESM1_ESM.pdf]

Supplementary Information

Molecular fingerprints resolve affinities of Rhynie Chert organic fossils

Loron, C.C., Rodriguez Dzul, E., Orr, P.J., Gromov, A.V., Fraser, N.C., McMahon, S.

1) Supplementary Figures.

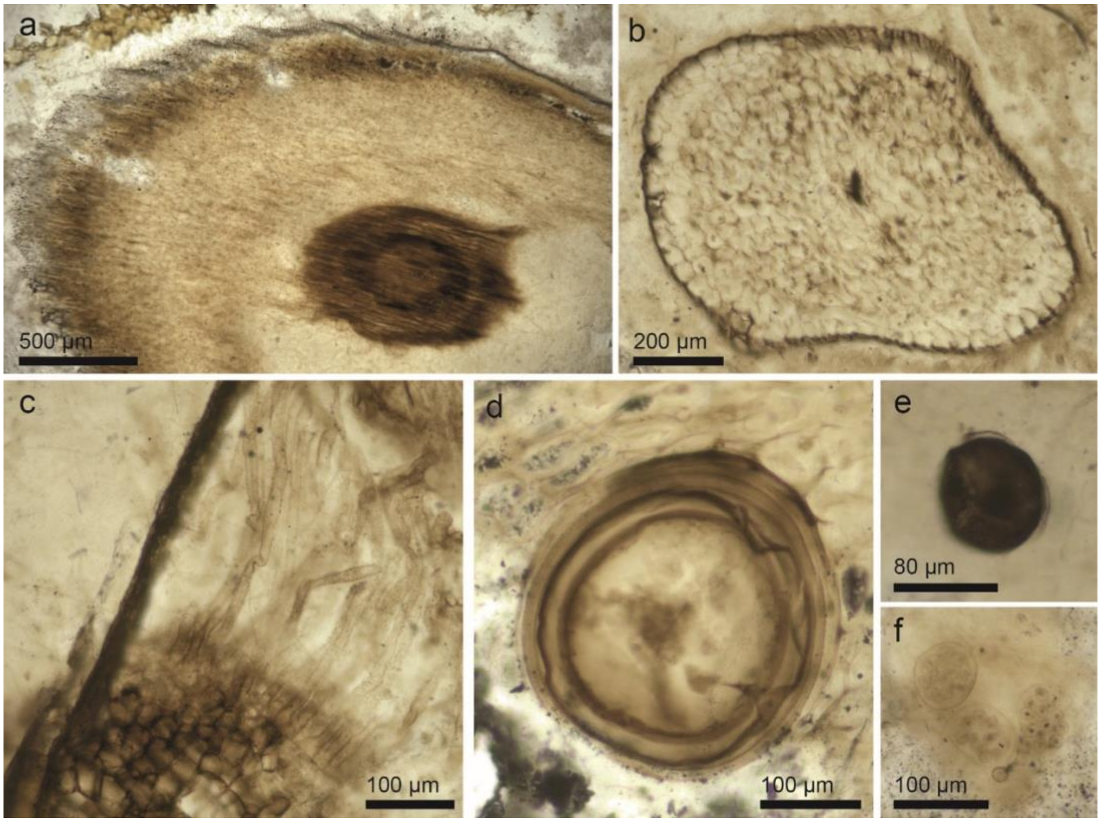

Supplementary Figure 1. Additional photomicrographs of representative Rhynie chert eukaryotic. *Aglaophyton majus* (plant, a); *Rhynia gwynne-vaughanii* (plant, b); Rhizoids (fungi, c); Glomeromycotan spore preserved in plant cortex (fungi, d); plant spore (e); *Frankbaronia polyspora* (peronosporomycetes, f).

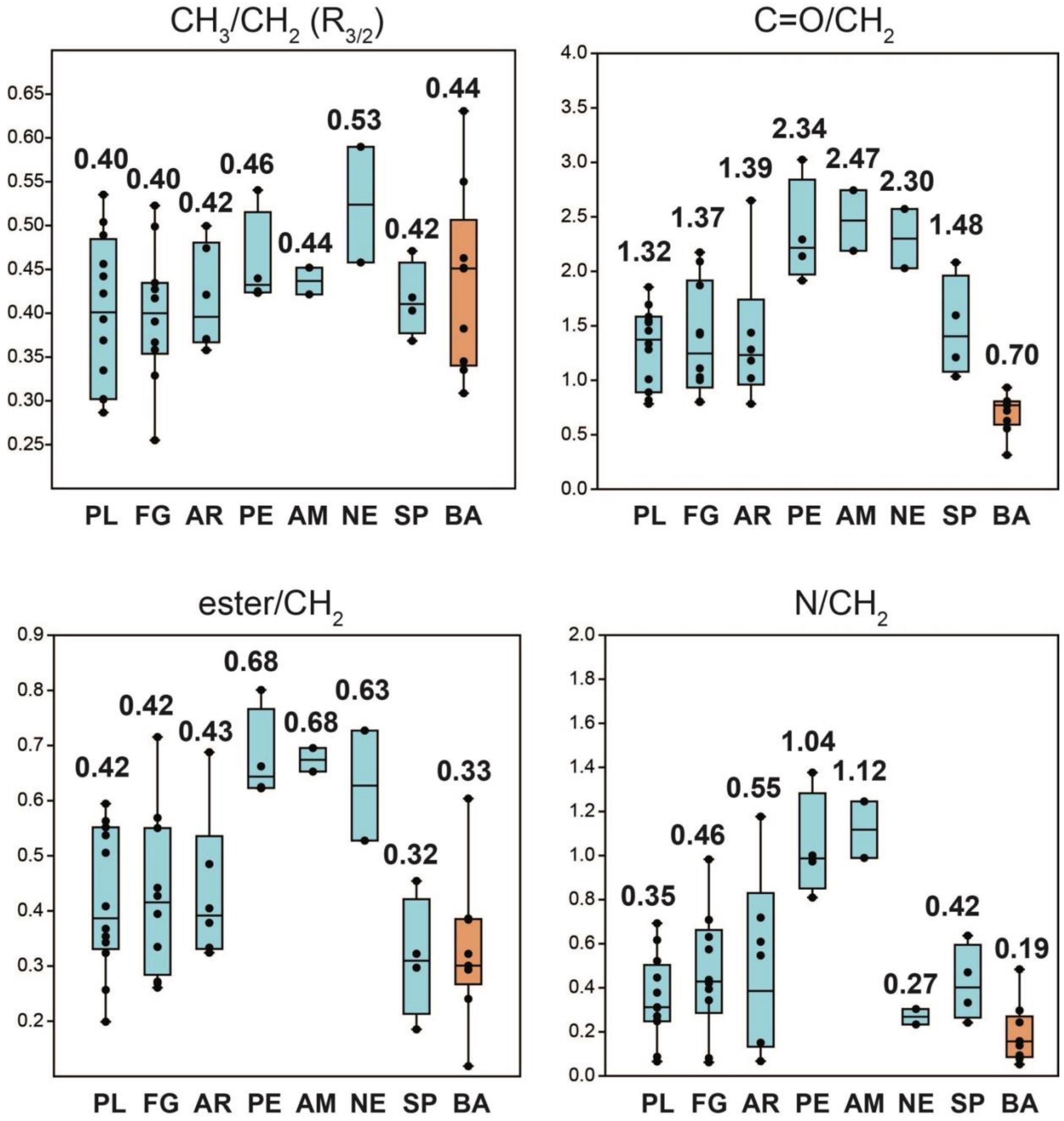

Supplementary Figure 2. Chemometric ratios of plants (**PL; n=12**); Fungi (**FG; n=10**); Arthropods (**AR; n=6**); Peronosporomycetes (**PE; n=4**); Amoebae (**AM; n=2**); Nematophytes (**NE; n=2**); Plant spores (**SP; n=4**); and Bacteria (**BA; n=9**). Blue boxes are eukaryote groups and orange are cyanobacteria. Each black dot represents one specimen. The whisker-plots show the lower and upper extreme (vertical black line), the first and third quartile (upper and lower box edges) and the median (horizontal black lines in boxes). Numbers are the mean value for each group. Source data are provided in the Source Data file.

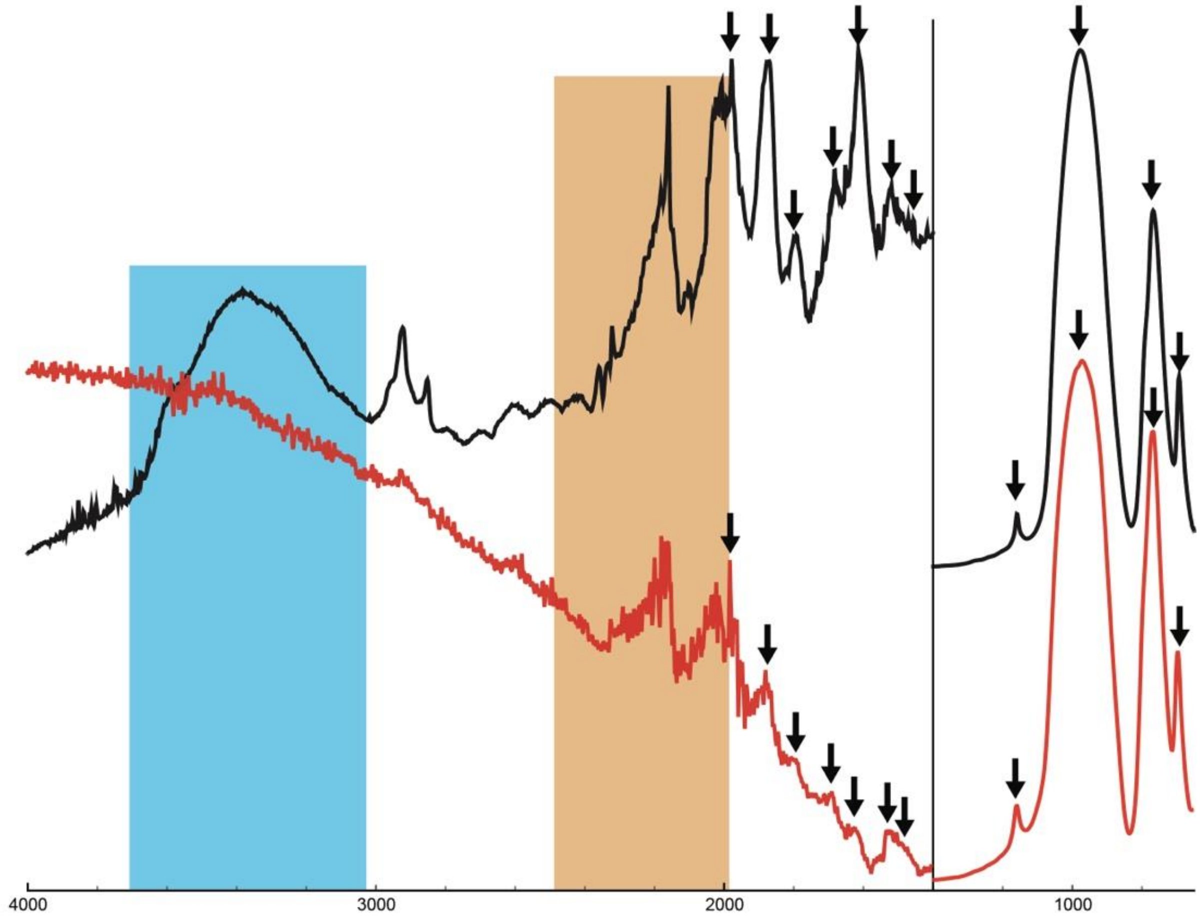

Supplementary Figure 3. Averaged raw spectra for the 49 analyzed Rhynie Chert specimens (black) and raw spectra for the chert matrix (red). Black arrows correspond to vibration bands associated with silica (Si-O); blue rectangle corresponds to the interval dominated by hydroxyl group vibration (various OH groups and H_2O in the chert). Brown rectangle corresponds to the interval dominated by atmospheric CO_2 vibration and artefact from the diamond coated ATR crystal.

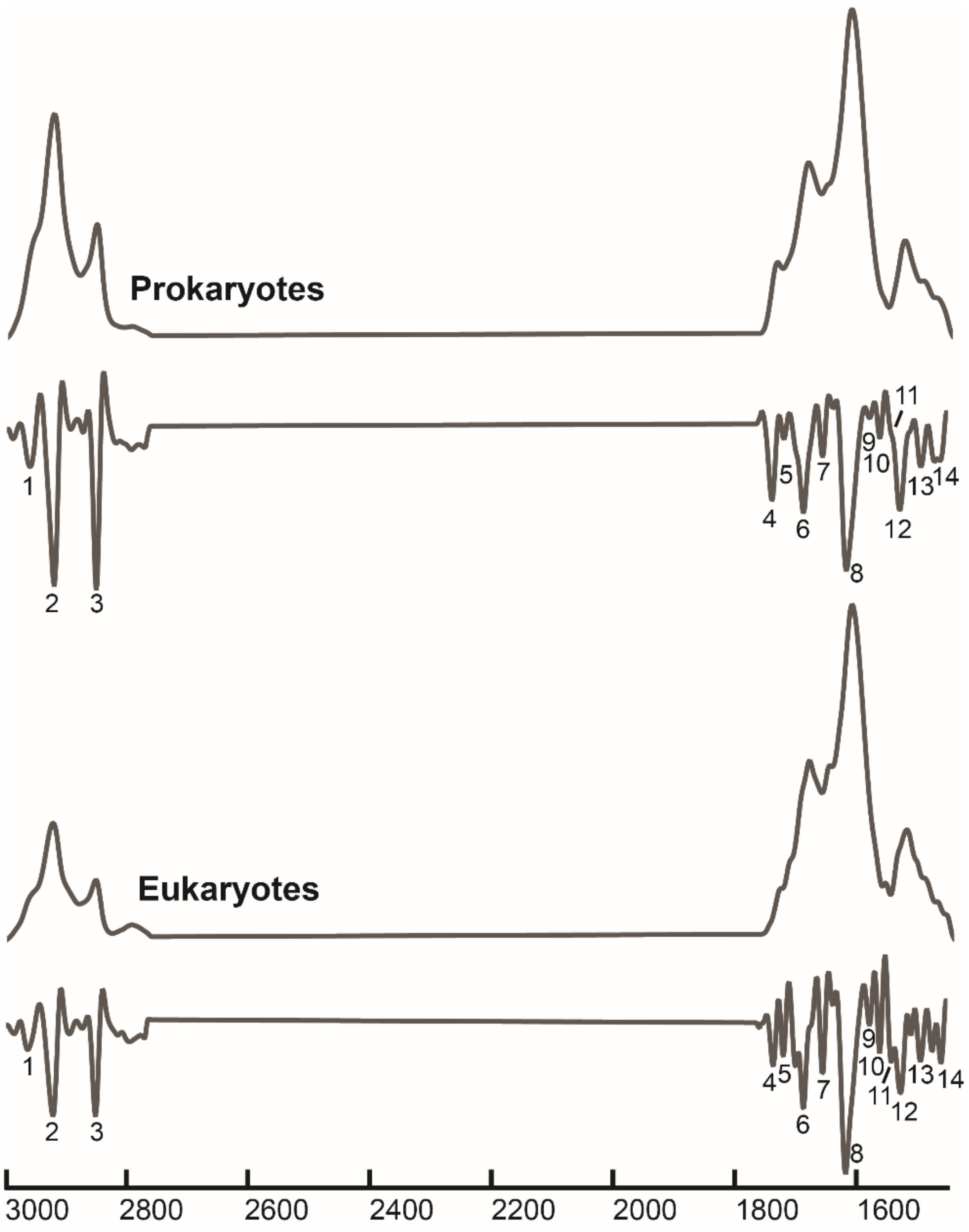

Supplementary Figure 4. Preprocessed averaged ATR-FTIR spectra and second derivative spectra for Prokaryote and Eukaryote organisms from the Rhynie Chert. Numbers on second derivative spectra correspond to band numbers in Supplementary Table 2.

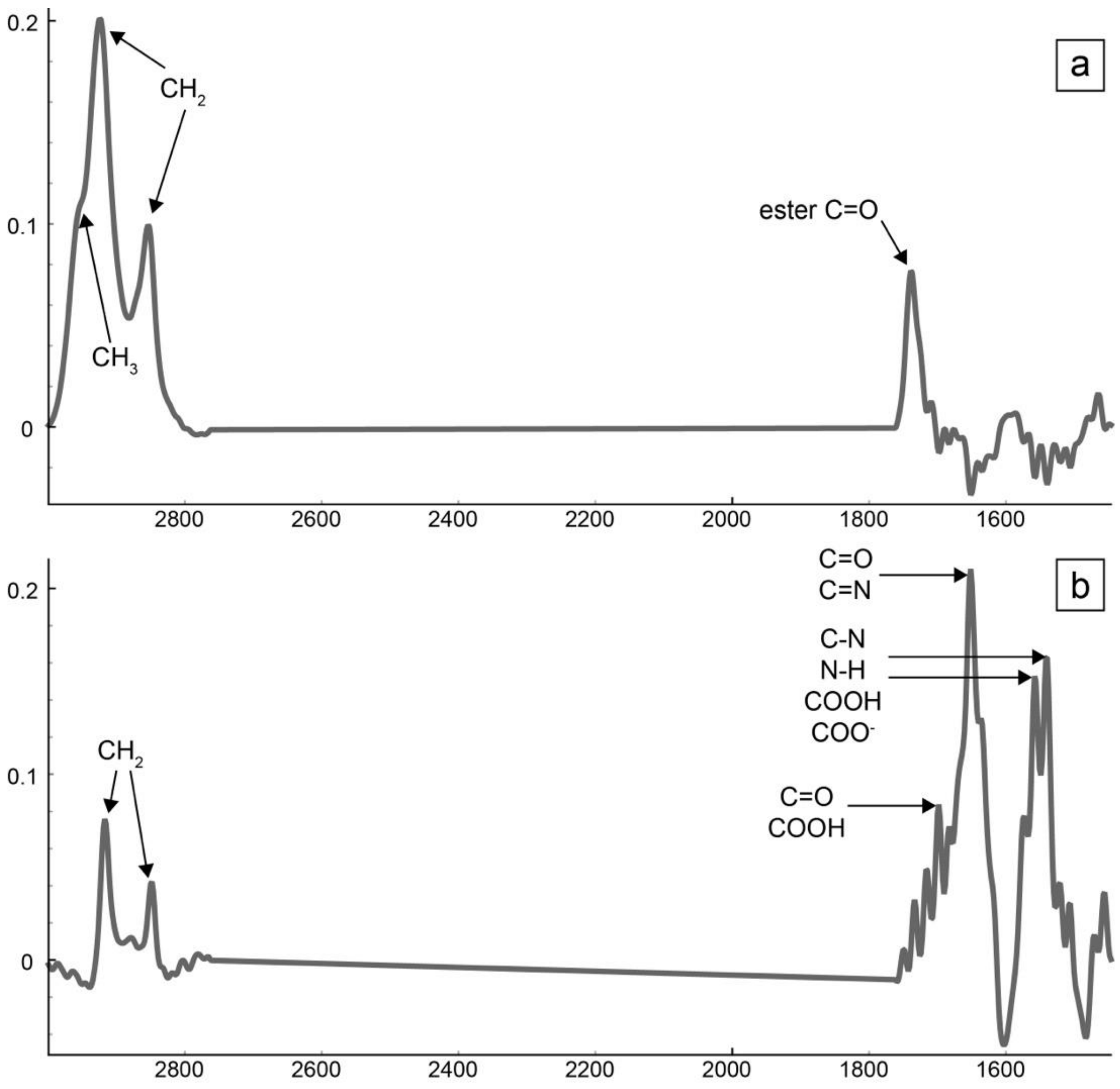

Supplementary Figure 5. PC spectral loadings. PC1 loading (A) shows a positive correlation with lipid moieties (aliphatic CH_x) and ester C=O groups. PC2 loading (B) shows a positive correlation with fossilization products of polysaccharides and proteins (COOH, C=O, N-moieties, COO^-) along with aliphatic CH2 groups. Source data are provided in the Source Data file.

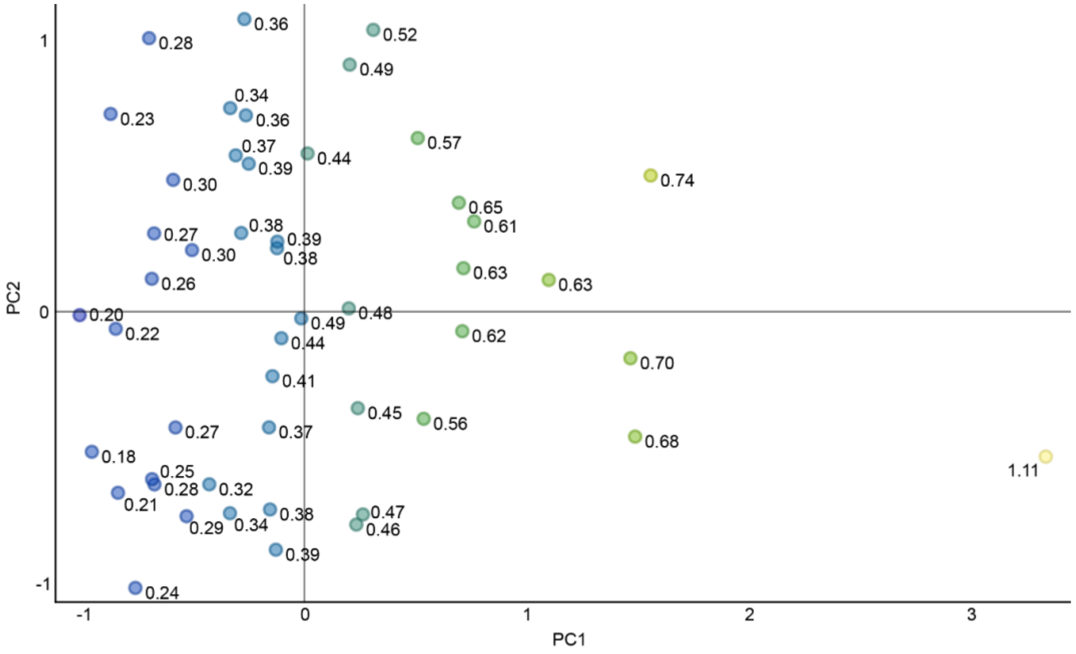

Supplementary Figure 6. PC score plot (PC1 vs PC2 as in main Figure 2a) showing values of CH_2/silica ratio. This ratio increases along PC1. Source data are provided in the Source Data file. The color gradient illustrates the increasing value of the ratio (from blue to yellow, rightward).

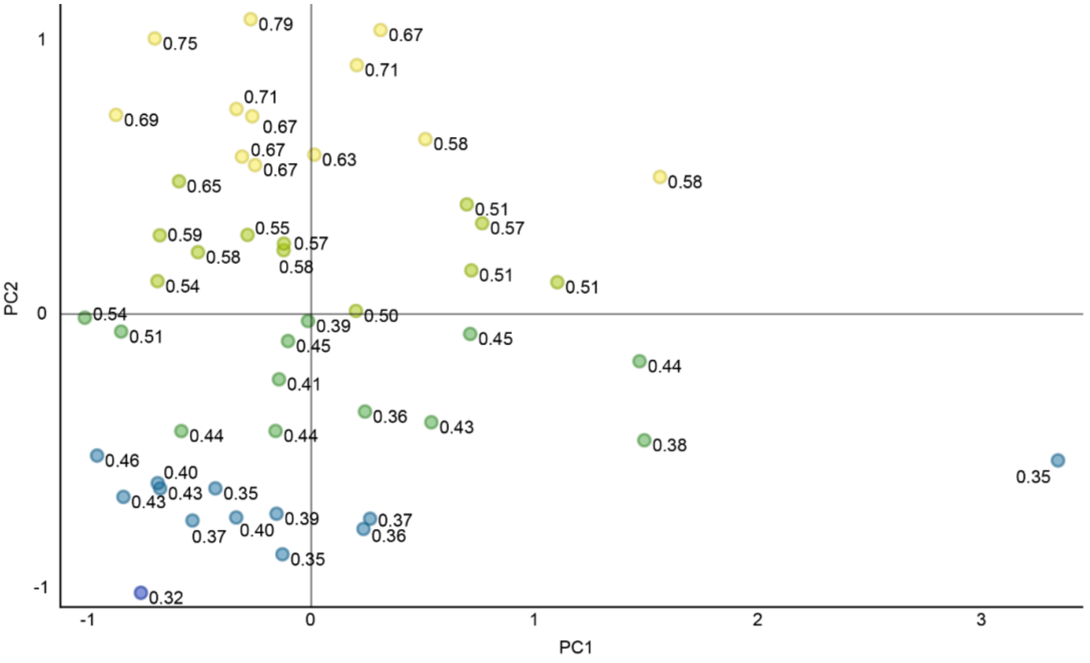

Supplementary Figure 7. PC score plot (PC1 vs PC2 as in main Figure 2a) showing values of C=O/silica ratio. This ratio does not increase along PC1, indicating that PC1 is tracking variations in the composition of, not the amount of, organic matter. Source data are provided in the Source

Data file. The color gradient illustrates the increasing value of the ratio (from blue to yellow, upward).

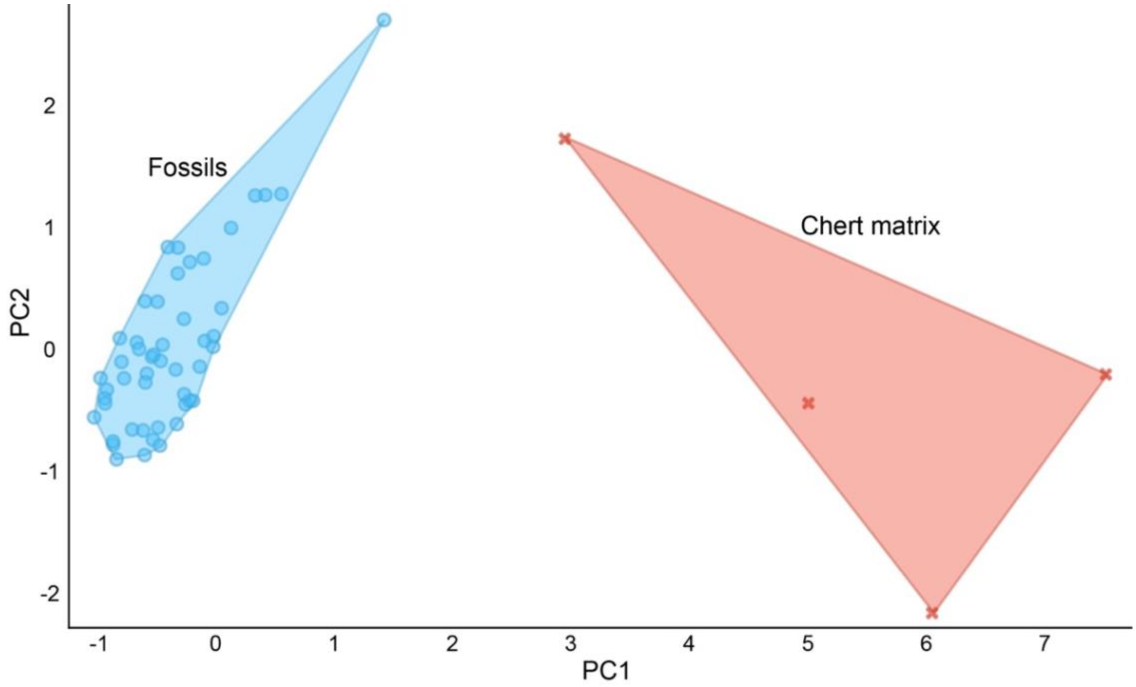

Supplementary Figure 8. Fossil endogeneity. PC1 vs PC2 score plot of principal component analyses of fossil material (blue) and chert matrix (red). Principal component 1 represents 67% of the variance and PC2, 15%. Source data are provided in the Source Data file.

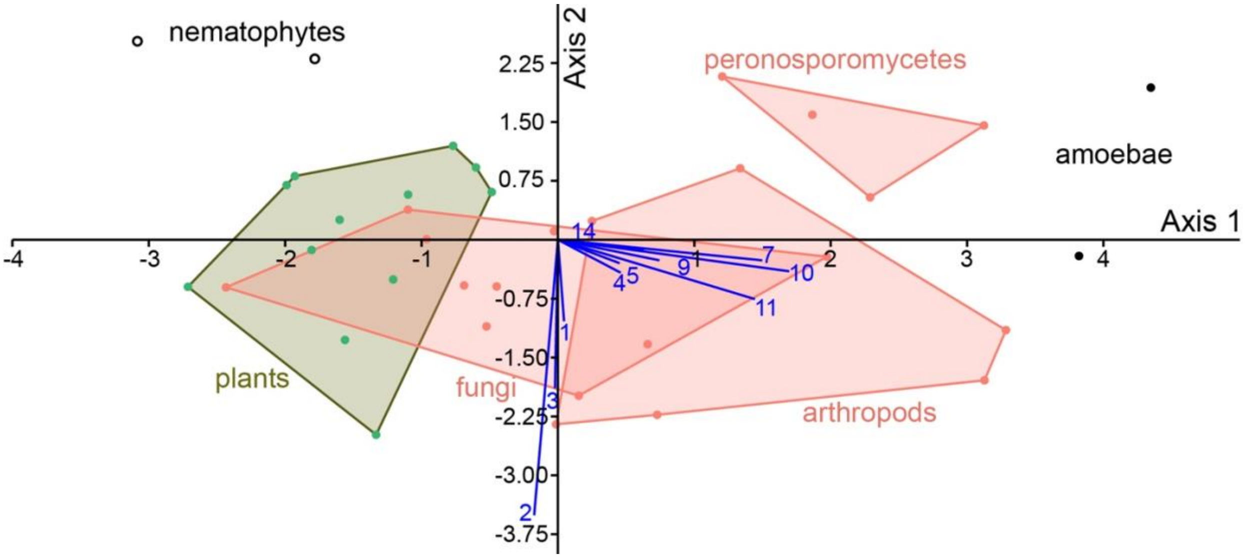

Supplementary Figure 9. Canonical Correspondence Analysis**.** Alternative presentation of main Figure 3. Discriminant factors are represented by numbered blue lines. Blue numbers correspond to specific absorption bands presented in Supplementary Data 2. Numbers 4, 5, 7, 9-11

correspond to absorption bands of sugar and protein fossilization products (C=O, COOH, COO^-, N-moieties); numbers 1-3, 14 correspond to absorption for lipids and aliphatic groups (14 is too small to be visible). Green hull correspond to fossil with an original composition depleted in amino-glucan sugar and red hulls correspond to fossil with an original composition enriched in amino-sugar. Source data are provided in the Source Data file.

2) Supplementary Tables

Supplementary Table 1. Band assignments. Numbers in first column correspond to band numbers as shown in Supplementary Figure 4.

Number	Band (cm^{-1})	Assignment	References
**1**	2961-2963	Asymmetric ν(CH_3)	[1]
**2**	2920-2923	Asymmetric ν(CH_2)	[1]
**3**	2851-2852	Symmetric ν(CH_2)	[1]
**4**	1732-1737	ν(C=O) ester	[1]
**5**	1717	ν(COOH); ν(C=O)	[1, 2, 3]
**6**	1684	Si-O	[4, 5]
**7**	1652-1654	ν(C=O); ν(C=N); (Amide I)	[1, 2, 3]
**8**	1614	Si-O	[4, 5]
**9**	1573-1575	ν(C=N); COOH; carboxylate	[1, 2, 6]
**10**	1557-1560	ν(C-N); δ(N-H); COOH; (Amide II)	[1, 2]
**11**	1540	ν(C-N); δ(N-H); COOH; carboxylate	[1, 2, 6]
**12**	1524	Si-O	[4, 5]
**13**	1490	Si-O	[4, 5]
**14**	1457-1462	δ(CH_2); δ(C-H)	[1, 2]

3) Supplementary Note

***Band Assignments*.** The use of ATR-FTIR allows study of the fossils in situ within the rocks without any alteration due to extraction and preparation of the organic material. In living organisms, C=O groups and amides will usually contribute the main absorption in the spectrum. As diagenesis progresses, with the carbonization and removal of heteroatoms, bands for this groups decrease in absorbance whereas absorption of C=C groups in aromatic structures increase (e.g.,[7,8,9]). However, degradation via peroxidation processes of polysaccharide and protein-rich tissues also yield various concentrations of oxygenated moities[10,11]. The main absorption bands in kerogenous fossils are often the contributions of the aromatic groups

represented by a strong band around 1600 cm^{-1} (conjugated C=C; often strongest of the spectrum) and several bands below 900 cm^{-1} (C-H out-of-plane bend)[12]. Due to the Si-O vibration[4,5], these two sets of bands are not observable in chert-preserved organisms. It is possible that the band at 1615 cm^{-1} in the Rhynie fossils represents the dual contribution of silica and aromatic groups. However, the absence of a marked aromatic C-H stretching band at ca. 3100 cm^{-1} as observed in mature kerogen (see fig. 1 in [8]) reflects a relatively low aromatic contribution in the present case.

The organic bands observed here, and retained by the PCA components, correspond to the expected bands for the fossilization products of sugar, protein and lipids. Degradation of polysaccharide-rich tissue produces carboxyl (COOH); carbonyl (C=O) and nitrogen-moieties (e.g., N-heterocycles)[10]. The bands observed at ca. 1735, 1717, 1650, 1575, 1560 and 1540 cm^{-1} (bands 4, 5, 7, 9, 10, 11 on Supplementary Figure 4 and Table 1) reflect the contribution of these fossilization products to the kerogen[1,3]. In addition, bands at 1575 and 1540 cm^{-1} also indicate a contribution of carboxylate groups (COO^-), a common function in degrading organic material[12] and in fossilization products of polysaccharide rich tissues at the silica interface[6,13]. Interactions taking place at the silica interface will also produce ester-SiO moieties in kerogen rich in carboxylate moieties[14].

4) Supplementary References

1. Coates, J. (2000). Interpretation of infrared spectra, a practical approach. In: Meyers, R.A., Ed., Encyclopedia of Analytical Chemistry, John Wiley & Sons Ltd., Chichester, 10881-10882
2. Movasaghi, Z., Rehman, S., & Rehman, D. I. (2008). Fourier transform infrared (FTIR) spectroscopy of biological tissues. *Applied Spectroscopy Reviews*, *43*(2), 134-179.
3. Mohsin, G. F., et al. (2018). Structural characterization of melanoidin formed from D-glucose and L-alanine at different temperatures applying FTIR, NMR, EPR, and MALDI-ToF-MS. *Food Chemistry*, *245*, 761-767.

4. Ito, Y., & Nakashima, S. (2002). Water distribution in low-grade siliceous metamorphic rocks by micro-FTIR and its relation to grain size: a case from the Kanto Mountain region, Japan. *Chemical Geology*, *189*(1-2), 1-18.

5. Igisu, M., et al. (2022). Spatial distribution of organic functional groups in Ediacaran acritarchs from the Doushantuo Formation in South China as revealed by micro-FTIR spectroscopy. *Precambrian Research*, *373*, 106628.

6. Lu, Y., & Miller, J. D. (2002). Carboxyl stretching vibrations of spontaneously adsorbed and LB-transferred calcium carboxylates as determined by FTIR internal reflection spectroscopy. *Journal of colloid and interface science*, *256*(1), 41-52.

7. Tissot, B. P., & Welte, D. H. (1984). From kerogen to petroleum. In *Petroleum formation and occurrence* (pp. 160-198). Springer, Berlin, Heidelberg.

8. Lis, G. P., Mastalerz, M., Schimmelmann, A., Lewan, M. D., & Stankiewicz, B. A. (2005). FTIR absorption indices for thermal maturity in comparison with vitrinite reflectance R0 in type-II kerogens from Devonian black shales. *Organic geochemistry*, *36*(11), 1533-1552.

9. Vandenbroucke, M., & Largeau, C. (2007). Kerogen origin, evolution and structure. *Organic Geochemistry*, *38*(5), 719-833.

10. McCoy, V. E., et al. (2020). Chemical signatures of soft tissues distinguish between vertebrates and invertebrates from the Carboniferous Mazon Creek Lagerstätte of Illinois. *Geobiology*, *18*(5), 560-565.

11. Wiemann, J., Crawford, J. M., & Briggs, D. E. (2020). Phylogenetic and physiological signals in metazoan fossil biomolecules. *Science advances*, *6*(28), eaba6883.

12. Painter, P. C., et al. (1981). Concerning the application of FT-IR to the study of coal: a critical assessment of band assignments and the application of spectral analysis programs. *Applied Spectroscopy*, *35*(5), 475-485.

13. Janssen, K., Mähler, B., Rust, J., Bierbaum, G., & McCoy, V. E. (2022). The complex role of microbial metabolic activity in fossilization. *Biological Reviews*, *97*(2), 449-465.

14. Hantal, G., Brochard, L., Dias Soeiro Cordeiro, M. N., Ulm, F. J., & Pellenq, R. J. M. (2014). Surface chemistry and atomic-scale reconstruction of kerogen–silica composites. *The Journal of Physical Chemistry C*, *118*(5), 2429-2438.
