# Supplementary material for: Molecular fingerprints resolve affinities of Rhynie chert organic fossils
Source: Nat Commun. 2023 Mar 13;14:1387. doi: 10.1038/s41467-023-37047-1 (PMC10011563; doi:10.1038/s41467-023-37047-1)
Supplement: Supplementary file 3 — Description of Additional Supplementary Files [file 41467_2023_37047_MOESM3_ESM.pdf]

Supplementary Data 1. List of specimens, coordinates, and repositories. Specimen codes: 2829p: Oxford University Museum of Natural History. Rfa, Rfe, Rff, Rfg, ALG2, AGLyon 2019, AGL127, Agl75 2019-1: University of Aberdeen. NMSRC9: National Museums of Scotland.
