# Supplementary material for: Molecular fingerprints resolve affinities of Rhynie chert organic fossils
Source: Nat Commun. 2023 Mar 13;14:1387. doi: 10.1038/s41467-023-37047-1 (PMC10011563; doi:10.1038/s41467-023-37047-1)
Supplement: Supplementary file 5 — Reporting Summary [file 41467_2023_37047_MOESM5_ESM.pdf]

nature portfolio

Corresponding author(s): Corentin Loron

Last updated by author(s): 13/02/2023

Reporting Summary

Nature Portfolio wishes to improve the reproducibility of the work that we publish. This form provides structure for consistency and transparency in reporting. For further information on Nature Portfolio policies, see our Editorial Policies and the Editorial Policy Checklist.

Statistics

For all statistical analyses, confirm that the following items are present in the figure legend, table legend, main text, or Methods section.

n/a	Confirmed	
☐	☒	The exact sample size (*n*) for each experimental group/condition, given as a discrete number and unit of measurement
☐	☒	A statement on whether measurements were taken from distinct samples or whether the same sample was measured repeatedly
☐	☒	The statistical test(s) used AND whether they are one- or two-sided<br>*Only common tests should be described solely by name; describe more complex techniques in the Methods section.*
☐	☒	A description of all covariates tested
☐	☒	A description of any assumptions or corrections, such as tests of normality and adjustment for multiple comparisons
☐	☒	A full description of the statistical parameters including central tendency (e.g. means) or other basic estimates (e.g. regression coefficient) AND variation (e.g. standard deviation) or associated estimates of uncertainty (e.g. confidence intervals)
☐	☒	For null hypothesis testing, the test statistic (e.g. F, t, r) with confidence intervals, effect sizes, degrees of freedom and P value noted<br>*Give P values as exact values whenever suitable.*
☒	☐	For Bayesian analysis, information on the choice of priors and Markov chain Monte Carlo settings
☒	☐	For hierarchical and complex designs, identification of the appropriate level for tests and full reporting of outcomes
☒	☐	Estimates of effect sizes (e.g. Cohen's *d*, Pearson's *r*), indicating how they were calculated

Our web collection on statistics for biologists contains articles on many of the points above.

Software and code

Policy information about availability of computer code

Data collection	Qual ID 2.51 (Smiths), Leica Application Suite 4.0
Data analysis	Quasar 1.6.0 (Orange-spectroscopy; Toplak et al., 2017; 2021), PAST 4.09 (Oyvind Hammer, Oslo University)

For manuscripts utilizing custom algorithms or software that are central to the research but not yet described in published literature, software must be made available to editors and reviewers. We strongly encourage code deposition in a community repository (e.g. GitHub). See the Nature Portfolio guidelines for submitting code & software for further information.

Data

Policy information about availability of data

All manuscripts must include a data availability statement. This statement should provide the following information, where applicable:

- Accession codes, unique identifiers, or web links for publicly available datasets
- A description of any restrictions on data availability
- For clinical datasets or third party data, please ensure that the statement adheres to our policy

Thin-sections are available from their respective collections: Specimen codes: 2829p: Oxford University Museum of Natural History. Rfa, Rfe, Rff, Rfg, ALG2, AGLyon 2019, AGL127, Agl75 2019-1: University of Aberdeen. NMSRC9: National Museums of Scotland. Raw spectra are available on Edinburgh DataShare at https://doi.org/10.7488/ds/3806.. Source data are provided as a Source Data file.

Human research participants

Policy information about studies involving human research participants and Sex and Gender in Research.

Reporting on sex and gender	NA
Population characteristics	NA
Recruitment	NA
Ethics oversight	NA

Note that full information on the approval of the study protocol must also be provided in the manuscript.

Field-specific reporting

Please select the one below that is the best fit for your research. If you are not sure, read the appropriate sections before making your selection.

☐ Life sciences ☐ Behavioural & social sciences ☒ Ecological, evolutionary & environmental sciences

For a reference copy of the document with all sections, see nature.com/documents/nr-reporting-summary-flat.pdf

Ecological, evolutionary & environmental sciences study design

All studies must disclose on these points even when the disclosure is negative.

Study description	Study of organic fossils preserved in a chert by attenuated reflection infra-red spectroscopy (ATR-FTIR)
Research sample	The samples are thin sections of chert (silica) from the Rhynie Chert, Aberdeenshire, Scotland containing fossils of plants, fungi, bateria and protists. The samples (fossils from the Rhynie chert) were chosen because they are exceptionally well-preserved, well-studied and characterized, and therefore provide a good positive control (fossils of known affinity based on morphology) for interpreting the observed FTIR signals.
Sampling strategy	The studied fossils were selected because they were well-preserved and present at the surface of the thin-sections. Sample size was not pre-determined but constrained by the occurrence of well preserved fossil material at the surface of the thin sections and by the need to eliminate confounding factors (e.g., by acquiring spectra from the same taxa in multiple thin sections).
Data collection	Optical microscopy was conducted on a Leica DM2700P at the UK Centre for Astrobiology (University of Edinburgh). Photographs were acquired in transmitted light using x10 and x20 objectives and the Leica Application Suite (4.0) software. Attenuated Total Reflectance-FTIR was conducted at room temperature on a Smiths IlluminateIR microscope equipped with liquid nitrogen cooled MCT detector providing spectral resolution of 4 cm-1, using a diamond coated ATR objective (magn x36) at the School of Chemistry (University of Edinburgh). Spectra were acquired in reflection mode for each specimen by combining 128 accumulations in the range 4000-650 cm-1 with an aperture of 100µm with the software Qual ID (Smiths).<br><br>C.C.L., S.M. and P.J.O. conceived the study. S.M., N.C.F., P.J.O. acquired and prepared samples. S.M., E.R. and C.C.L. acquired photomicrographs. C.C.L. and A.V.G. acquired the ATR data. C.C.L. processed the data and conducted multivariate and supervised analyses with input from S.M. and E.R. C.C.L. and S.M. drafted the manuscript and figures. All authors reviewed and edited the manuscript.
Timing and spatial scale	Data acquisitions were conducted between February 2022 and October 2022. Fossils were located in thin sections made from rock wafers about 2 cm x 2 cm x 0.1 mm. Spectra were acquired by combining 128 accumulations with an aperture of 100µm
Data exclusions	No data were excluded
Reproducibility	We analysed nine fossil cyanobacteria, six different arthropod cuticles, four different plant spores, eleven plant cortices (Aglaophyton majus, Rhynia gwynne-vaughanii) and one set of rhizoids, four Peronosporomycota , two amoebozoan, ten fungi and two tubular nematophytes. The eukaryotes are in nine rock thin sections obtained from two different sample collections, and the cyanobacteria are in four rock thin sections obtained from three different sample collections. Spectra were also acquired from the silica matrix.
Randomization	The biological affinities and species names of the studied specimens are based on the descriptions by previous researchers on the Rhynie Chert. Based on these descriptions the samples were allocated into Eukaryotes and Prokaryotes for the supervised analyses and into Fungi, Arthropods, Plants, Peronosporomycetes, Amoebae and Nematophytes for the descriptive LDA.
Blinding	NA

Did the study involve field work? ☐ Yes ☒ No

Reporting for specific materials, systems and methods

We require information from authors about some types of materials, experimental systems and methods used in many studies. Here, indicate whether each material, system or method listed is relevant to your study. If you are not sure if a list item applies to your research, read the appropriate section before selecting a response.

Materials & experimental systems

n/a	Involved in the study
☒	☐ Antibodies
☒	☐ Eukaryotic cell lines
☐	☒ Palaeontology and archaeology
☒	☐ Animals and other organisms
☒	☐ Clinical data
☒	☐ Dual use research of concern

Methods

n/a	Involved in the study
☒	☐ ChIP-seq
☒	☐ Flow cytometry
☒	☐ MRI-based neuroimaging

Palaeontology and Archaeology

Specimen provenance	407-Ma Rhynie Chert fossil assemblage of Aberdeenshire, Scotland. Permits were not required as museum specimens were used. The specimens ultimately come from a small number of digs carried out by university and museum palaeontologists with permission of the landowners over the past century. Since the Rhynie Chert does not crop out there is no other means of accessing the material.
Specimen deposition	The samples are deposited in the Oxford University Museum of Natural History, the University of Aberdeen and the National Museums of Scotland Rhynie Chert collections.
Dating methods	No new dates are provided

☐ Tick this box to confirm that the raw and calibrated dates are available in the paper or in Supplementary Information.

Ethics oversight	No ethical approval was required (study of fossils within rock thin-sections)

Note that full information on the approval of the study protocol must also be provided in the manuscript.
